# A Possible Role for Pioglitazone in the Management of Depressive Symptoms in Metabolic Syndrome Patients (EPICAMP Study): A Double Blind, Randomized Clinical Trial 

**DOI:** 10.1155/2014/697617

**Published:** 2014-08-25

**Authors:** Hamidreza Roohafza, Pedram Shokouh, Masoumeh Sadeghi, Zahra Alikhassy, Nizal Sarrafzadegan

**Affiliations:** ^1^Isfahan Cardiovascular Research Center, Cardiovascular Research Institute, Isfahan University of Medical Sciences, Isfahan, P.O. Box 81465-1148, Iran; ^2^Isfahan Cardiac Rehabilitation Research Center, Cardiovascular Research Institute, Isfahan University of Medical Sciences, Isfahan, P.O. Box 81465-1148, Iran

## Abstract

The present trial aimed to evaluate the effects of pioglitazone on the mental status of nondiabetic metabolic syndrome patients. From 145 patients screened, 104 eligible volunteers (57% female; age 20–70 years) were enrolled and randomly assigned to receive either pioglitazone (uptitrated to 30 mg/day; *P* = 53) or matching placebo (*P* = 51) for 24 weeks. Depression and anxiety were quantified using the hospital anxiety and depression scale and stress level using the general health questionnaire 12 at baseline, week 12, and endpoint. Homeostasis model assessment was used to estimate insulin resistance. At week 24, pioglitazone was superior in mitigating depression score (*P* = 0.011). In trend analysis, the effect of time (*P* < 0.001) and group (*P* = 0.023) as well as the time by group interaction (*P* = 0.032) on the mean depression score was considerable. In contrast, significant decrements in anxiety and stress levels (*P* < 0.001 and *P* = 0.012, resp.) were comparable between two groups. With respect to our findings, alterations in depression severity were not correlated with changes in insulin resistance level (*P* = 0.145). In conclusion, our findings suggest that pioglitazone might be able to improve mood in nondiabetic insulin resistant patients. (Registered at Australian New Zealand Clinical Trials Registry; ACTRN12611000351910.)

## 1. Introduction

A chronic, progressive, and complex metabolic disturbance, the metabolic syndrome (MetS), is closely related to serious cardiovascular and emotional complications [1, 2]. Its increasing prevalence worldwide [3] has pushed the MetS more and more under the spotlight during the last decades. Incontrovertibly, unlike the cardiometabolic aspects, the level of our knowledge regarding the accompanying psychological problems with this syndrome is still in its primitive stages.

So far, the significance of the relationship between MetS and a wide variety of psychological disturbances has been verified. Preliminary data points to a positive relationship between the MetS and chronic work stress [4]. Similar associations have also been reported with generalized anxiety disorder [5] and anxiety symptoms [6] but remained controversial according to the findings of others [2, 7]. However, during the last years, much focus of investigators has been placed on the relativity of this syndrome and depressive symptoms. In spite of being contradictory [7, 8] and not confirming a causal relationship, the available evidence concerning the association of depressive symptoms and the MetS could not be neglected [2, 9, 10]. The considerable point is that the most consistent findings were yielded from the analysis of the construct of MetS than of its component parts [11]. More surprisingly, this association seems to be a two-way street as the core components of the MetS could also precipitate the development of depression [6, 12], which suggests a shared biological pathology.

Although the monoamine theory has still remained the predominant framework for understanding depression, it faces major constraints in explaining some clinical observations such as the delayed action and the considerable rate of treatment failure with conventional monoaminergic antidepressants [13]. The association of depressive symptoms with metabolic impairments of the MetS further emphasizes the insufficiency of this theory. Trying to complement the monoamine theory, roles of several potential mediating mechanisms have been investigated, among which the hypothalamic-pituitary axis dysregulation, insulin resistance (IR), immune-inflammatory activation, and oxidative stress have gained more priority [8, 14, 15]. The practical conclusion of this argument is to try to verify the effect(s) of anti-inflammatory and/or insulin-sensitizing agents on psychological status of MetS patients. To date, a broad spectrum of insulin-sensitizing agents has been studied in the treatment of psychiatric disorders with promising results [15]. Among them, much attention has been devoted to a group of structurally related peroxisome proliferator-activated receptor gamma (PPAR*γ*) agonists, the thiazolidinediones (TZDs).

PPAR*γ* is a transcription factor that regulates numerous genes involved in the regulation of cell differentiation and metabolism [16]. Agonists of this receptor have shown effectiveness in improving IR in nondiabetic MetS patients [17]. As a member of the TZDs family, pioglitazone is an effective insulin sensitizer with considerable anti-inflammatory, neuroprotective, and antiexcitotoxic properties [18] that makes it a good candidate to be tried to improve mood in insulin resistant individuals.

This paper is presenting the findings of psychiatric part of the EPICAMP (effects of pioglitazone on cardiac structure, markers of endothelial function, and psychological status) study, a trial aimed at investigating the efficacy of pioglitazone monotherapy in improving a number of cardiovascular and psychiatric indices in a group of nondiabetic MetS patients. The results of the other part of this study have been reported previously [19]. The purpose of designing this part was to elucidate the effects of pioglitazone on the mental status of the participants. Our primary endpoints were to assess the degree of changes in depression, anxiety, and stress scores in the intervention group compared with the placebo group.

## 2. Materials and Methods

### 2.1. Study Design

The present study was conducted following the principles of a double-blind placebo-controlled trial. Consented participants were equally assigned to receive either pioglitazone (uptitrated to 30 mg/day; *n* = 53) or matching placebo (*n* = 51) for 24 weeks. Randomized allocation was carried out using sequentially numbered boxes and was decided at the time of admission. The random number list was generated by a random-list generator software. None of the staff who randomized the patients had role in data collection and analysis. For more confidentiality, the codes were used to address the patients at all of the follow-up phases. Pioglitazone and matching placebo tablets were provided by Osvah Pharmaceutical Company (Tehran, Iran). Participants were receiving their usual medical treatments during the follow-up period.

The study protocol was approved by the Isfahan Cardiovascular Research Institute ethics committee, affiliated to the Isfahan University of Medical Sciences as well as the ethics committee of the AJA University of Medical Sciences, Tehran, Iran. Study aims and methods were verbally explicated to all participants and a written consent was obtained prior entering the screening phase. The present trial was registered in the Australian New Zealand Clinical Trials Registry (http://www.anzctr.org.au, identifier: ACTRN12611000351910) and Iranian Registry of Clinical Trials (http://www.irct.ir, identifier: IRCT201101023733N2).

### 2.2. Study Population

Potential participants were randomly selected from the electronic database of the first phase of Isfahan Healthy Heart Program. This program was a comprehensive integrated community-based study whose details are presented elsewhere [20]. Selected patients were invited to our clinic in the Isfahan Cardiovascular Research Institute from March to May of 2011 to undergo screening laboratory tests as well as physical examination and a structured interview by a physician. Individuals were marked eligible if they had MetS and were not diabetic. MetS was diagnosed following the harmonized criteria using the indices of obesity for Middle Easterns [21]. The diagnosis of type 2 diabetes mellitus was made based on the American Diabetes Association diagnostic criteria [22]. Our major exclusion criteria were as follows: New York Heart Association functional class 3 or 4, any history or evidences of ischemic heart disease, impaired liver function (alanine transaminase >2.5 times of upper limit of normal), renal dysfunction with serum creatinine >1.5 mg/dL, any debilitating medical condition, participation in a weight reduction program, being under treatment with a TZD within the preceding three months, having any of the pioglitazone contraindications, pregnancy, and lactation.

### 2.3. Efficacy and Safety Assessments

The anthropometric variables of eligible volunteers were measured according to standard methods by a trained nurse by means of a calibrated scale and a stadiometer. Blood pressure was measured in the right arm at sitting position after a 10-minute rest using a standard mercury sphygmomanometer. The body mass index was calculated using the formula: weight (kg)/height^2^ (m^2^). All demographic variables were registered in preprepared questionnaires. Clinical structured interview and examination were done by a single trained physician at baseline, midpoint (week 12), and endpoint. Three standard self-administered questionnaires were filled under the supervision of a single psychologist and superintended by an expert psychiatrist. Lifestyle modification recommendations were offered to all patients at every visit including dietary control and regular exercise training (at least 30 min/day 3–5 days a week) advice. A fasting blood sample was also obtained from each patient at every visit. Telephone-based interviews at weeks 6 and 18 were structured and carried out by the same physician. At midpoint of the trial, all patients were invited to have a general physical examination, psychological interview, and blood sampling. At study end, similar clinical, anthropometric, and laboratory variables to the baseline were measured. All telephone-based and face-to-face interviews were done using a preprepared checklist which was designed to assess the patients' adherence to medication as well as to find any plausible side effects of pioglitazone, specially the congestive heart failure, with high sensitivity. To ensure the patients' safety, liver transaminases were monitored every 3 months during the follow-up course. If the occurred side effects were not indications for withdrawal, the administered medication was downtitrated by half. Our interviewer was continually making sure that all women of childbearing potential were using an approved method of birth control throughout the study.

In order to quantify the changes in depression and anxiety level throughout the study period the hospital anxiety and depression scale was used [23, 24], which is a 14-item, self-administered scale consisting of two subscales, anxiety and depression (seven items each). Every item scores on a four-point scale (0–3) corresponding to an increasing severity of symptoms, which makes an overall score range of 0 to 21 for either of the subscales. We accounted the depression and anxiety scores as continuous quantitative variables in all of the analyses except for the calculation of number needed to treat (NNT) for depression, in which a cut point of 8/21 or higher was used to determine a treatment response. The general health questionnaire 12 was used in the estimation of the stress level of our patients. This instrument is a widely used screening instrument for detecting psychological distress in the general population [25]. Each item scores on a 0-0-1-1 method (less than usual, no more than usual, fairly more than usual, and much more than usual) which produces an overall score ranging from 0 to 12 with higher scores, more severe distress.

### 2.4. Laboratory Assays

Blood specimens were obtained at baseline, midpoint, and endpoint after at least 12 hours of overnight fasting. Samples were placed into tubes containing ethylene diamine tetra acetic acid and were centrifuged to separate the serum which was used to analyze the biochemical factors. Serum alanine transaminase and aspartate transaminase levels were measured by Hitachi 902 auto-analyzer (Japan) using Pars Azmoon (Iran) analytical kits. Serum fasting glucose measurement was done using the same machine and Biosystem (France) kits. Serum insulin levels were assayed by enzyme-linked immunosorbent assay (ELISA) method using Monobind kits (CA, USA). Insulin sensitivity in the fasting state was assessed with homeostasis model assessment (HOMA) and calculated using the formula: fasting plasma glucose (milligrams per deciliter) × fasting serum insulin (microunits per milliliter)/405, as originally described by Matthews et al. [26]. All the measurements were carried out in Isfahan Cardiovascular Research Institute Laboratory which is qualitatively controlled by National Reference Lab of Iran (Tehran, Iran) and INSTAND e.V. Laboratory (Düsseldorf, Germany).

### 2.5. Statistical Analysis

Baseline demographic data are presented as mean ± SD or number (percent), where appropriate. As there were withdrawals from both groups, we conducted the analyses based on the intention-to-treat principles, which included all patients who had at least one postbaseline efficacy assessment. The significance of the effect of time and group on the changes of the primary outcome variables in repeated measurements was studied using linear mixed model analysis in linear mixed-effects model. For each assessment, the net score reduction was defined as a separate variable. Pair-wise comparisons between two groups were done using independent-sample *t*-test for quantitative and chi-square test for discreet variables. The Pearson correlation test was also used to examine the correlation between the changes of depression and HOMA-IR scores. The significance of differences in the rate of side effects was determined by Fisher's exact test. We used statistical package for Social Sciences software version 15.0 (SPSS Inc., Chicago, Illinois, USA) in analyzing the data. A 2-tailed *P* value of ≤0.05 was considered significant in all of the analyses.

## 3. Results

**Figure 1:**
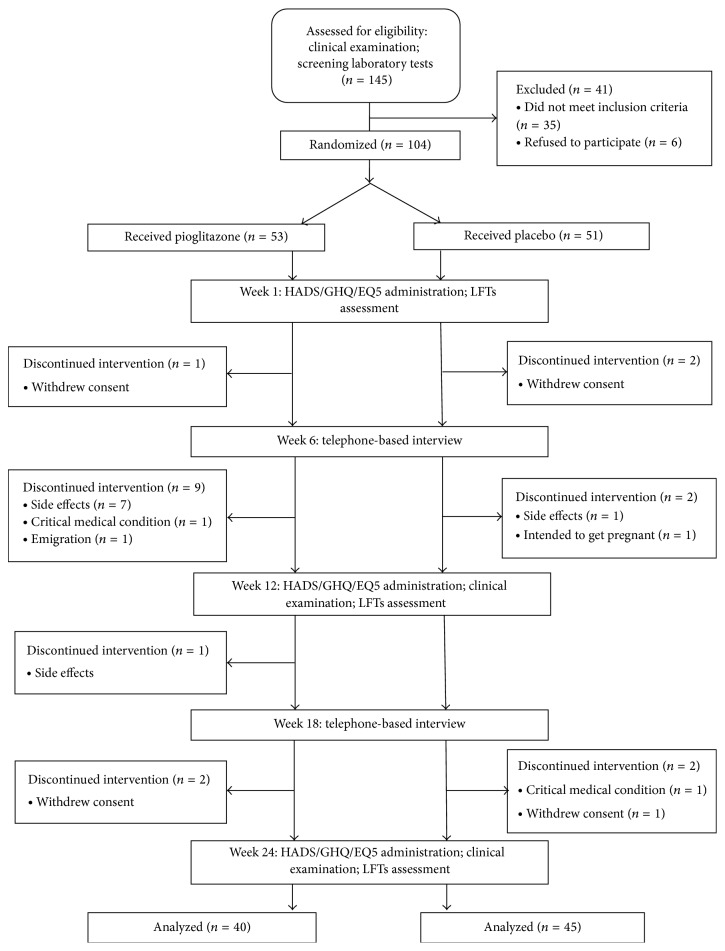
Study flow diagram.

145 randomly selected individuals were invited to undergo a screening process which led to the selection of a final sample of 104 eligible volunteers (57% female; age range: 20–70 years old). The baseline characteristics of our intent-to-treat study population are summarized in Table 1. As shown, there was no considerable difference between the two study groups regarding demographics, smoking status, lipid profile, and IR. Furthermore, the two groups showed similar concomitant use of various classes of antidepressants at baseline and during the intervention period. According to the study flow diagram presented in Figure 1, 41 individuals were excluded through the screening phase, owing to either not being eligible or refusing to participate. There were also dropouts before the time of the first efficacy assessment (4 participants from the placebo and 11 from the intervention group).

### 3.1. Efficacy Measures

Pair-wise analyses of mean scores in each time point are briefed in Table 2. Using independent-sample *t*-test, the mean value of depression score was shown to be significantly lower only in pioglitazone group at week 24 compared with the placebo group (*P* = 0.011). As it could be noticed from Table 2, none of the other outcome measures' values were considerably different between groups.

**Figure 2:**
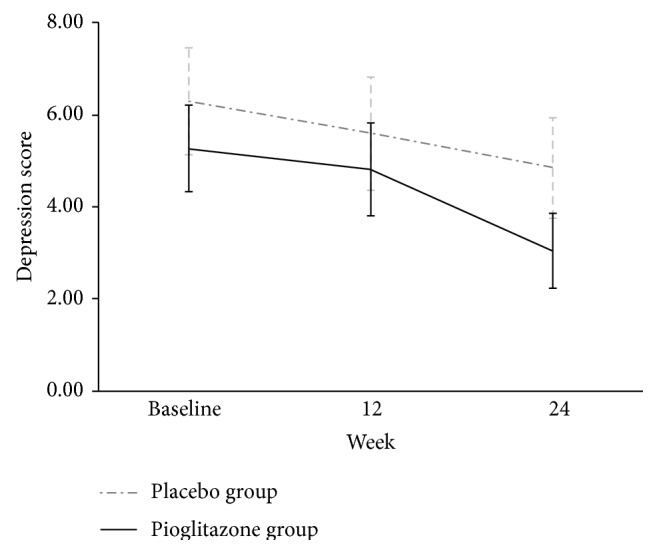
The comparison of the trend of changes in depression score over time in pioglitazone versus placebo groups (data are mean scores ± SD).

In trend analysis of changes, depression severity was found to change considerably over time (*Z* = −4.91; *P* < 0.001) and between groups (*Z* = −2.00; *P* = 0.023). In addition to these effects, a significant time by group interaction (*Z* = −1.92; *P* = 0.032) shows that the pioglitazone-induced changes over time were different from that of the placebo. The analysis of trend in individual groups also produced significant values (*P* < 0.001 in pioglitazone and *P* = 0.006 in placebo group).  Figure 2 displays these trends of changes in a more presentable form. The number needed to treat for depression in our intend-to-treat population was 5.8 (95% CI: 4.9–7.2).

According to our analysis, a significant change in anxiety score over repeated assessments (*Z* = −4.28; *P* < 0.001) and in each group (*P* < 0.001) was recognized, while inconsiderable results were produced regarding the effect of group (*Z* = −1.66; *P* = 0.096). Similarly, stress score was diminished significantly during the time course of the trial (*Z* = −2.52; *P* = 0.012) and in the placebo group (*P* < 0.002) but the between- roup difference never reached a significant level (*Z* = −1.22; *P* = 0.222).

In pioglitazone-assigned patients, HOMA-IR score was markedly diminished (*Z* = −1.89; *P* = 0.047) while between group factor was insignificant. The changes of HOMA-IR score in pioglitazone group were not correlated with the changes in depression score (*r* = 0.144; *P* = 0.145). It is worth noting that our findings regarding the indices of glucose and lipid metabolism have been described in detail elsewhere [19].

### 3.2. Safety and Tolerability

Although no serious adverse event was reported, there were withdrawals from both groups caused by intervention-related side effects (Figure 1). New onset or aggravated symptoms in pioglitazone and placebo groups are listed and compared in Table 3. Although not being significantly more frequent, most side effects have only been reported in the intervention group. It should be noted that this research might not be powered enough to analyze the difference. Symptoms which caused the participants to abandon the study were as follows: peripheral edema (1 case), dizziness (2 cases), headache (1 case), tiredness (1 case), cardiac arrhythmia (2 cases), and nausea (1 case). There was one case of alanine transaminase rise above 2.5 times of the upper limit of normal after 3 months of pioglitazone administration, who was excluded from the study.

## 4. Discussion

The present trial was designed in order to provide evidence regarding the influence of pioglitazone on different aspects of psychological status under the conditions of IR. Our findings represent a pronounced declining trend in the level of depression, anxiety, and stress in the participants of both groups. We suppose that the given lifestyle modification recommendations and being under observation as well as a placebo-effect had resulted in such parallel meliorations. Despite being quite modest in degree, the net difference between endpoint depression scores and the time-by-group interaction effect was statistically significant and might suggest a pharmacologic efficacy of the intervention. On the other hand, a number needed to treat 5.8 in a general population of MetS patients shows that almost 17% of affected individuals would benefit psychologically from the pioglitazone treatment, which is an appreciable advantage. However, in-detail discussion around the clinical significance of these findings is out of the scope of this paper.

When it comes to the antidepressant efficacy of TZDs, what merits highlighting is the consistency of all of the available studies conducted either in animal or clinical settings [27–33]. Pioglitazone and rosiglitazone were shown to be efficacious in decreasing depression severity in insulin resistant depressed patients in two 12-week open-label pilot studies [29, 30]. Evidence of a higher quality has recently become available, produced by two randomized double-blind trials in metabolically normal depressed individuals [33] and obese patients with polycystic ovarian syndrome [28].

It has been shown that antidepressant effect of TZDs is mediated through PPAR*γ* receptors [27, 31] but what that has sparked intense debate is the pathway(s) through which PPAR*γ* receptors act. Theoretically, the modification of IR is the first candidate to put the blame on. It has been suggested that insulin has a substantial part in modulation of the neuronal concentration of neuropeptides and monoamines [34]. Additionally, a positive correlation between IR and the severity of depressive symptoms has been found in cross-sectional studies [35]. Nonetheless, the limited available clinical studies have not produced coherent results. In contrast with what has been observed in a pilot study [29], we failed to find a positive correlation between depression score decrement and the improvement of IR. Apart from that, the between group changes of HOMA-IR score were not significant and could not serve a mechanistic explanation for the recorded antidepressant response in pioglitazone group. Our findings are corroborated by two reports by Rasgon et al. [30] and Kashani et al. [28]. More significantly, the antidepressant efficacy of pioglitazone in a group of insulin-sensitive patients [33] does point to the involvement of biological mechanisms beyond simple insulin sensitization, while it is not powered to reject the role of central nervous system IR.

Currently, there is scientific ground for considering a role for inflammatory processes in the pathophysiology of depression. Not only the elevated levels of proinflammatory cytokines are frequently reported in MDD patients, but also a positive coherence was found between proinflammatory activation and the severity of depressive symptoms [36]. Therefore, it could be hypothesized that anti-inflammatory agents could, at least partly, contribute to the normalization of neuronal function in MDD. This hypothesis was supported by a recently published open-label trial [37]. According to a growing body of evidence, TZDs exhibit anti-inflammatory properties through the modulation of different levels of inflammatory response. PPAR*γ* agonists are shown to significantly inhibit macrophage-inflammatory response through suppressing the secretion of several cytokines and reducing the expression of inducible nitric oxide synthase [38]. Neuroprotective capacities of TZDs are not confined to alleviating inflammation, as they support neuronal survival through upregulating of Bcl-2 antiapoptotic protein [39] and attenuating quinolinic acid induced neurotoxicity in animals [40]. Other functions of PPAR*γ* agonists that have been put forward as mediators of their antidepressant-like activity are the reduction of N-methyl-D-aspartate mediated calcium currents in hippocampal neurons [32] and modulation of Wnt signaling components [41]. The marked antioxidant properties of TZDs [42] also deserve consideration, as the redox balance in depressed individuals is reported to be disturbed [43] and an antioxidant agent, n-acetyl cysteine, has shown efficacy in the treatment of depressive symptoms in bipolar disorder [44]. Overall, it seems that the observed antidepressant effect of TZDs is likely to be explicated best through more than a single physiologic pathway.

When taking the effects of PPAR*γ* agonists on anxiety and stress into consideration, reaching a conclusion is much more difficult as little comparable evidence is available. We failed to trace any higher anxiolytic efficacy of pioglitazone than placebo, while Kumar et al. have reported a significant reduction in anxiety-like behavior in mice after receiving moderate to high doses of pioglitazone for three weeks [45]. Regarding the effects of TZDs on stress level, our findings would not confirm the previously reported observations with rosiglitazone in rats. This member of the TZDs family has been shown to be efficient in blunting the systemic responses to acute psychological stress [46] and restoring neuronal glucose metabolism and brain ATP levels after exposure to stress [47].

The present trial benefitted from having a double-blind and placebo-controlled design, as well as a reasonable course of follow-up and a randomly selected sample, which discriminates it from the similar trials [28–30, 33]. Nonetheless, some issues should be considered when generalizing the results. Firstly, our study was aimed at the general population of nondiabetic MetS patients and not merely at ones with anxiety disorder or MDD, so our findings might not be applicable to these subgroups. Secondly, the considerable dropout rate before the first assessment would decrease the power of the study to detect differences. Another factor that theoretically might influence the validity of our data is using of self-report questionnaires. And finally the use of HOMA-IR technique for estimating IR could also be considered a limitation. Although HOMA-IR estimations have shown a good correlation with those obtained by hyperglycemic and euglycemic clamps [26], their power to measure the whole body and central nervous system IR remained undetermined.

## 5. Conclusion

The findings of the present study offer evidence that pioglitazone, in conjunction with its promoting effects on insulin sensitivity, is able to significantly improve the mood of nondiabetic MetS patients. This data might be useful in weighing up the risks and benefits of adding pioglitazone to the drug regimen of this group of patients. However, being preliminary, our findings await confirmation from larger studies.

## Figures and Tables

**Table 1:** Baseline characteristics of intent-to-treat study population.^a^

	Placebo (*n* = 45)	Pioglitazone (*n* = 40)	*P* value
Male sex	16 (35.6%)	19 (47.5%)	.264
Age (years)	51.34 ± 9.29	51.57 ± 9.53	.909
Education			.411
Undergraduate	39 (86.7%)	36 (90.0%)	
Graduate	6 (13.3%)	4 (10.0%)	
Current smoker^b^	4 (7.8%)	10 (18.9%)	.108
Waist circumference (cm)	101.74 ± 10.17	102.50 ± 9.15	.719
Blood pressure (mmHg)			
Systolic	134.23 ± 21.13	130.67 ± 15.94	.409
Diastolic	85.87 ± 10.18	83.24 ± 8.43	.223
Fasting glucose (mg/dL)	94.13 ± 11.04	97.97 ± 12.92	.149
Serum lipids (mg/dL)			
Total cholesterol	216.07 ± 39.92	206.22 ± 31.23	.226
HDL-C	43.19 ± 8.62	41.70 ± 10.25	.478
LDL-C	119.35 ± 30.12	111.75 ± 20.81	.206
Triglycerides	201.75 ± 107.29	220.86 ± 141.53	.502
Fasting insulin (*μ*U/mL)	13.44 ± 7.96	14.13 ± 6.04	.688
HOMA-IR index	3.19 ± 2.02	3.33 ± 1.39	.748
Depression	15 (33.3%)	18 (40.9%)	.297
Psychiatric medications use	5 (11.1%)	4 (10.0%)	.678

^a^Data are expressed as mean ± SD for continuous variables and number (percentage) of participants for categorical variables.

^b^Defined as one who regularly smoked at least one cigarette per day.

**Table 2:** The pair-wise and trend analyses of changes in assessed variables in pioglitazone and placebo groups in repeated measurements.

Variable (mean ± SD)		Placebo	Pioglitazone	*P* value
Depression score	Baseline	6.29 ± 4.23	5.26 ± 3.47	.177
	Week 12	5.59 ± 4.47	4.81 ± 3.75	.339
	Week 24	4.84 ± 3.96	3.04 ± 3.02	**.011**
	*P* value	**0.006**	**<.001**	

Anxiety score	Baseline	7.25 ± 4.55	6.23 ± 4.07	.227
	Week 12	4.86 ± 4.79	4.43 ± 4.87	.652
	Week 24	3.75 ± 3.30	2.91 ± 2.85	.168
	*P* value	**<.001**	**<.001**	

Stress score	Baseline	2.76 ± 2.87	1.87 ± 2.48	.091
	Week 12	1.82 ± 2.57	1.55 ± 2.18	.555
	Week 24	1.45 ± 2.29	1.13 ± 1.70	.421
	*P* value	**.002**	.098	

**Table 3:** New onset or aggravated symptoms recorded during the follow-up period.

Symptom [number of cases (%)]	Placebo	Pioglitazone	*P* value
Peripheral edema	6 (11.8)	8 (15.1)	.556
Dizziness	0 (0)	4 (7.5)	.116
Headache	0 (0)	3 (5.7)	.241
Tiredness	0 (0)	2 (3.8)	.494
Cardiac arrhythmia	0 (0)	2 (3.8)	.494
Dyspnea on exertion	2 (3.9)	0 (0)	.494
Nausea	0 (0)	1 (1.9)	.999
